# Comprehensive Transcriptomic and Proteomic Analyses Identify a Candidate Gene Set in Cross-Resistance for Endocrine Therapy in Breast Cancer

**DOI:** 10.3390/ijms231810539

**Published:** 2022-09-11

**Authors:** Chung-Liang Li, Sin-Hua Moi, Huei-Shan Lin, Ming-Feng Hou, Fang-Ming Chen, Shen-Liang Shih, Jung-Yu Kan, Chieh-Ni Kao, Yi-Chia Wu, Li-Chun Kao, Ying-Hsuan Chen, Yi-Chen Lee, Chih-Po Chiang

**Affiliations:** 1 Department of Surgery, Kaohsiung Medical University Hospital, Kaohsiung Medical University, Kaohsiung 80756, Taiwan; 2 Division of Breast Oncology and Surgery, Department of Surgery, Kaohsiung Medical University Hospital, Kaohsiung Medical University, Kaohsiung 80756, Taiwan; 3 Graduate Institute of Medicine, Kaohsiung Medical University, Kaohsiung 80756, Taiwan; 4 Center of Cancer Program Development, E-Da Cancer Hospital, I-Shou University, Kaohsiung 82445, Taiwan; 5 Department of Biomedical Science and Environmental Biology, Kaohsiung Medical University, Kaohsiung 80756, Taiwan; 6 Graduate Institute of Clinical Medicine, Kaohsiung Medical University, Kaohsiung 80756, Taiwan; 7 Department of Surgery, Division of Plastic Surgery, Kaohsiung Medical University Hospital, Kaohsiung Medical University, Kaohsiung 80756, Taiwan; 8 Department of Anatomy, School of Medicine, College of Medicine, Kaohsiung Medical University, Kaohsiung 80756, Taiwan; 9 Department of Medical Laboratory Sciences and Biotechnology, Fooyin University, Kaohsiung 83102, Taiwan

**Keywords:** breast cancer, endocrine therapy resistance, cross-resistance, selective estrogen receptor modulators (SERMs), selective estrogen receptor degraders (SERDs), aromatase inhibitors (AIs), The Cancer Genome Atlas (TCGA)

## Abstract

Endocrine therapy (ET) of selective estrogen receptor modulators (SERMs), selective estrogen receptor downregulators (SERDs), and aromatase inhibitors (AIs) has been used as the gold standard treatment for hormone-receptor-positive (HR+) breast cancer. Despite its clinical benefits, approximately 30% of patients develop ET resistance, which remains a major clinical challenge in patients with HR+ breast cancer. The mechanisms of ET resistance mainly focus on mutations in the ER and related pathways; however, other targets still exist from ligand-independent ER reactivation. Moreover, mutations in the ER that confer resistance to SERMs or AIs seldom appear in SERDs. To date, little research has been conducted to identify a critical target that appears in both SERMs/SERDs and AIs. In this study, we conducted comprehensive transcriptomic and proteomic analyses from two cohorts of The Cancer Genome Atlas Breast Invasive Carcinoma (TCGA-BRCA) to identify the critical targets for both SERMs/SERDs and AIs of ET resistance. From a treatment response cohort with treatment response for the initial ET regimen and an endocrine therapy cohort with survival outcomes, we identified candidate gene sets that appeared in both SERMs/SERDs and AIs of ET resistance. The candidate gene sets successfully differentiated progress/resistant groups (PD) from complete response groups (CR) and were significantly correlated with survival outcomes in both cohorts. In summary, this study provides valuable clinical implications for the critical roles played by candidate gene sets in the diagnosis, mechanism, and therapeutic strategy for both SERMs/SERDs and AIs of ET resistance for the future.

## 1. Introduction

Breast cancer is the most common cancer in women worldwide with approximately 2.3 million incident cases (11.7%) in 2020. In the United States, breast cancer is the leading cancer type, accounting for 30% of new cases and 15% of death among all cancer types in women [1]. Breast cancer is a highly heterogeneous cancer that is classified based on the histopathology of the receptor status or microarray analysis of molecular subtypes [2]. With immunohistochemical staining, breast cancer is classified as hormone-receptor-positive based on the expression of the estrogen receptor (ER) and progesterone receptor (PR). In contrast, breast cancer without the expression of ER, PR, and human epidermal growth factor receptor-2 (HER2) is classified as triple-negative breast cancer (TNBC) [3]. According to the current guidelines, the classification is considered positive if at least 1% of the tumor nuclei stain for the receptor with appropriate internal and external controls [4]. Nearly 80% of breast cancers are estrogen-receptor-positive (ER+) or hormone-receptor-positive [2].

Estrogen is a critical hormone that is not only responsible for normal growth and development of female mammary and reproductive organs, but also mammary hyperplasia and tumorigenesis. When the estrogen receptor binds to estrogen, it dimerizes and is translocated to the nucleus with coactivators to activate gene transcription, including cell cycle progression [5,6,7]. Due to the high dependence of breast tumorigenesis on the estrogen-ER axis, endocrine therapy with estrogen suppression or ER antagonists is the first-line treatment for ER+ breast cancer. Clinically, endocrine therapy includes: selective ER modulators (SERMs), such as tamoxifen, which competitively inhibit the binding of estrogen to ER; selective ER downregulators (SERDs), such as fulvestrant, which impair mobility/translocation of ER; aromatase inhibitors (AIs), such as letrozole, which deplete systemic estrogen levels in post-menopausal patients by blocking the conversion of androgens to estrogen. The above molecules of endocrine therapy are approved for adjuvant treatment of ER+ breast cancer patients and can reduce the mortality rate of breast cancer by 30% [8,9]. 

Despite the clinical benefit of endocrine therapy, approximately 20–30% of patients acquire resistance and recurrence after long-term treatment with endocrine therapy [10,11,12,13]. The mechanisms of endocrine resistance are complex and mainly focus on gain-of-function mutations in ER and compensatory cross-talk between ER and growth factor receptor/oncogenic signaling pathways [9]. Based on clinical observations, mutations in the ligand-binding domain (LBD) of ESR1 (the gene encoding ERα) [9,14] and receptor tyrosine kinases of HER2 amplification [15] are predominantly observed in endocrine therapy resistance. In addition, mutations in oncogenic pathways such as PI3-AKT/MAPK pathways are also frequently observed in mutation profiles and endow endocrine resistance in breast cancer [9].

Endocrine resistance remains a major clinical challenge for therapeutic efficacy in most ER+ breast cancer patients. Besides the above mechanisms, there may still be other genetic targets of endocrine resistance and this could be a potential therapeutic strategy to overcome endocrine resistance. Moreover, endocrine resistance is commonly driven by ligand-independent ER reactivation [9,16]. The dominant mutation of ESR1 accounts for less than 20% of cases of endocrine resistance in patients treated with SERMs or AIs [9,17], which indicates that patients resistant to SERMs with ESR1 mutation may also not benefit from AIs. However, ESR1 mutations that confer resistance to SERMs or AIs seldom appear in SERDs [14]. To date, little research has been conducted to identify a critical target that appears in both SERMs/SERDs and AIs. Moreover, a previous study from The Cancer Genome Atlas (TCGA) mainly focused on studies of untreated primary tumors or datasets without treatment response [18,19,20,21,22]. Therefore, in this study, we aimed to comprehensively explore the critical target by integrating transcriptomic (RNA-seq) and proteomic (reverse-phase protein array, RPPA) approaches that appeared in both SERMs/SERDs and AIs from the TCGA dataset of two cohorts with endocrine therapy response and survival outcomes, and elucidating their potential in diagnostic and survival outcomes, thereby providing genetic profiles and strategies to overcome endocrine therapy resistance.

## 2. Results

### 2.1. Characteristics of Study Subjects from the TCGA-BRCA

In this study, 670 samples with ET were collected from TCGA-BRCA of the Genomic Data Commons (GDC) data portal. Based on the record of treatment response, most of the samples were recorded with “Not Applicable” or “Not Available”, and only 44 samples were recruited in the arm of the treatment response cohort (TR cohort) with the record of “Complete Response (CR)” and “Clinical Progressive Disease (PD)”. The 44 samples were further divided into two groups: CR = 34 samples and PD = 10 samples. The PD group showed clinical progress and resistance to the first-line ET regimen of AI (N = 5 samples) and SERM/SERD (N = 5 samples). The baseline characteristics of the study population groups according to CR and PD are summarized in Table 1. There were significant differences in the distribution of metastasis status, stage, PFS, and OS between CR and PD.

We aimed to integrate the dataset of transcriptomic (RNA-Seq) and proteomic (reverse-phase protein array, RPPA) analyses, which appeared in both SERMs/SERDs and AIs. The baseline characteristics of the treatment response cohort (CR and PD) were further divided into two tables, the RNA-seq and RPPA datasets, as shown in Table 2 and Table 3. Of the 44 samples, 32 possessed an RPPA dataset with CR = 25 and PD = 7, as shown in Table 3. Both tables show significant differences in the distribution of the proportion of stage, recurrence/metastases, and survival status between CR and PD. In addition, the baseline characteristics of the endocrine therapy cohort (ET cohort, without treatment response, N = 449) derived from the previous 670 samples are also included in Table 2 and Table 3.

### 2.2. The Critical Target in Both SERMs/SERDs and AIs of PD Groups 

**Figure 1:**
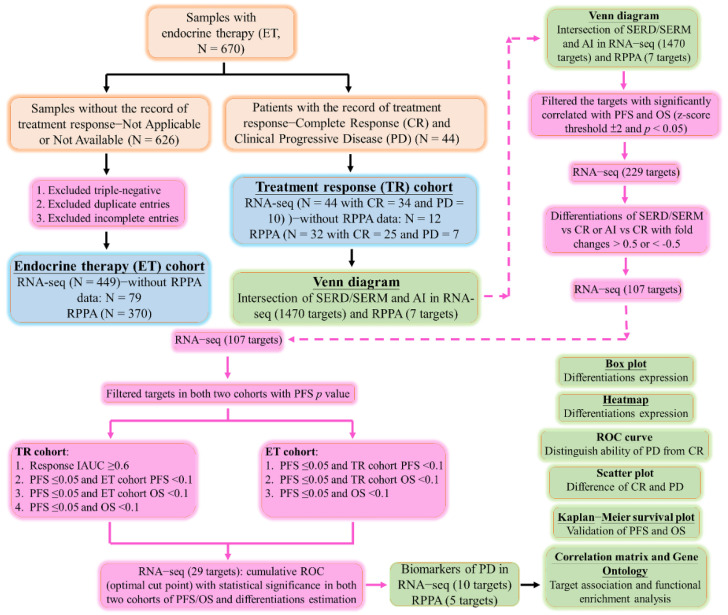
The workflow of the study.

**Figure 2:**
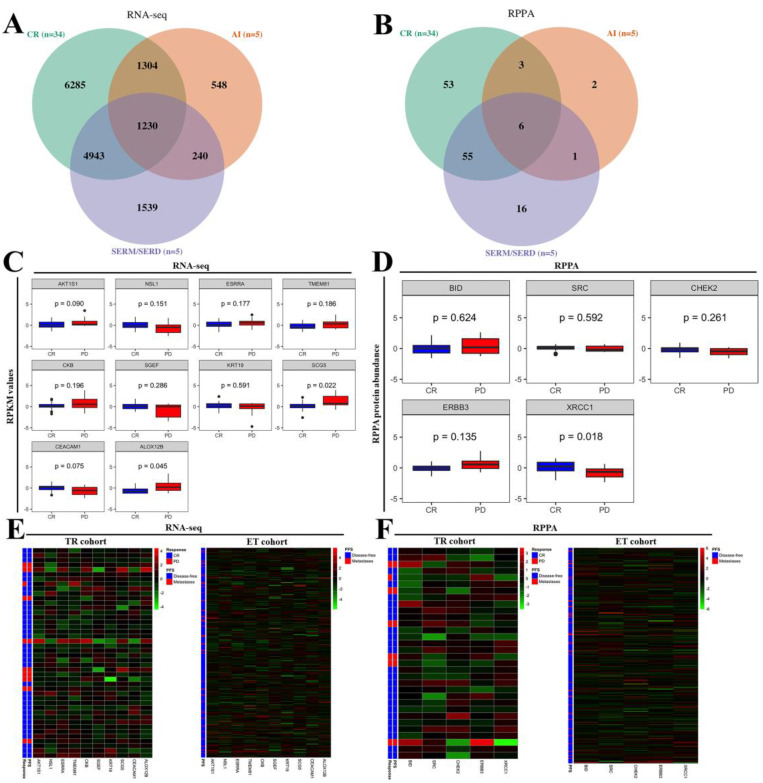
The candidate gene set of RNA−seq and RPPA in both SERM/SERD and AI of ET resistance. (**A**,**B**) The Venn diagram analysis shows that there are 1470 targets of RNA−seq and seven targets of RPPA in both SERM/SERD and AI of ET resistance. (**C**–**F**) Based on a series of analyses and selection, the expression of candidate gene sets from RNA−seq of ten targets and RPPA of five targets is displayed as box plots in the treatment response cohort (TR cohort) and heatmap in the treatment response (TR cohort) and endocrine therapy cohort (ET cohort).

To verify the critical target of transcriptomic and proteomic analyses in both SERMs/SERDs and AIs of PD groups, a Venn diagram was conducted in both RNA-seq and RPPA from the treatment response cohort. In the Venn diagram, the targets from each group of CR, AIs, and SERMs/SERDs were selected based on the criteria of z-score threshold ±2 in the TCGA database. A comprehensive workflow to display the screening and analysis approaches in this study is shown in Figure 1. There were 1470 mRNA targets that appeared in the region of overlap between SERMs/SERDs and AIs and among CR, SERMs/SERDs, and AIs (Figure 2A). In addition, seven protein targets appeared in the region of overlap between SERMs/SERDs and AIs and among CR, SERMs/SERDs, and AIs (Figure 2B). The seven protein targets were retained for further study; however, the 1470 mRNA targets were filtered through a series of workflows, as shown in Figure 1. We filtered 1470 targets with statistical significance for both PFS and OS, and obtained 229 targets (significantly correlated with both PFS and OS with the criteria of z-score threshold ±2 and *p* < 0.05). Then, these 229 targets were filtered with differentiations of SERD/SERM versus CR or AI versus CR with fold changes > 0.5 or < −0.5, and 107 targets were obtained. Subsequently, 107 targets were filtered in both the treatment response cohort (with ET treatment response) and the endocrine therapy cohort (with survival outcomes) based on progression-free survival (PFS). In the arm of the TR cohort, inclusion criteria were: (1) response of area under the curve (AUC) for CR vs. PD ≥0.6; (2) PFS ≤0.05 and PFS ≤0.1 in ET cohort; (3) PFS ≤0.05 and OS ≤0.1 in ET cohort; (4) both PFS ≤0.05 and OS ≤0.1. In the arm of the ET cohort (without treatment response of CR and PD), inclusion criteria were: (1) PFS ≤0.05 and PFS ≤0.1 in TR cohort; (2) PFS ≤0.05 and OS ≤0.1 in TR cohort; (3) both PFS ≤0.05 and OS ≤0.1. Finally, we obtained 29 targets from two cohorts with the clinical characteristics of both treatment response and survival outcomes.

The 29 targets from RNA-seq and seven targets from RPPA were calculated based on optimal cut-off points and individual receiving operating characteristics (ROC) analysis. The results of RNA-seq are shown in Table 4, which lists the cut-off points of high risk in each of the 29 targets. When the values reach the cut-off point of high risk, they receive a score of one; otherwise, a score of zero is accorded. The score was calculated in the total population of 44 samples for each target to obtain the results of AUC for distinguishing PD from CR (individual AUC, shown in the column of response) and AUC for PFS and OS (individual AUC, shown in the column of PFS, OS, and based on the criteria of optimal cut-off points). The above analyses were also conducted on targets from RPPA (Table 5). Finally, based on PFS, we filtered and arranged 29 targets with inclusion criteria: (1) response AUC ≥ 0.6 and PFS ≤ 0.05 in both TR and ET cohorts (*AKT1S1*, *NSL1*, *ESRRA*, *TMEM81*, *CKB*, *SGEF*); (2) statistical significance in PFS/OS of two cohorts (*KRT19*); (3) borderline significance between CR and PD (*SCG5*, *CEACAM1*, *ALOX12B*) and seven targets with inclusion criteria; (4) statistical significance in PFS of TR or ET cohort. Further cumulative ROC analysis was conducted based on the above criteria to obtain the optimal gene set (RNA-seq with ten targets and RPPA with five targets) of cumulative ROC values for response AUC and PFS AUC. The differentiations of candidate genes (RNA-seq with ten targets and RPPA with five targets) are summarized in Table 6 and Table 7.

### 2.3. The Distinguishability of Candidate Gene Sets in Identifying PD versus CR

Based on a series of analyses and selections, candidate genes of transcriptomics (RNA-seq with 10 targets) and proteomics (RPPA with five targets) were verified as critical gene sets that appeared in both SERMs/SERDs and AIs of ET resistance. The differentiations of RNA-seq expression with 10 targets in the treatment response cohort is illustrated in Figure 2C with a box plot. *AKT1S1*, *SCG5*, *CEACAM1*, and *ALOX12B* reached borderline significance between the CR and PD groups (*p* = 0.022 to 0.09). On the other hand, the differences in RPPA expression of five targets in the treatment response cohort is illustrated in Figure 2D with box plots. *XRCC1* reached a significant difference between the CR and PD groups (*p* = 0.018). Furthermore, the expression of candidate gene sets (RNA-seq and RPPA) in the treatment response and endocrine therapy cohort is shown with a heatmap in Figure 2E,F.

**Figure 3:**
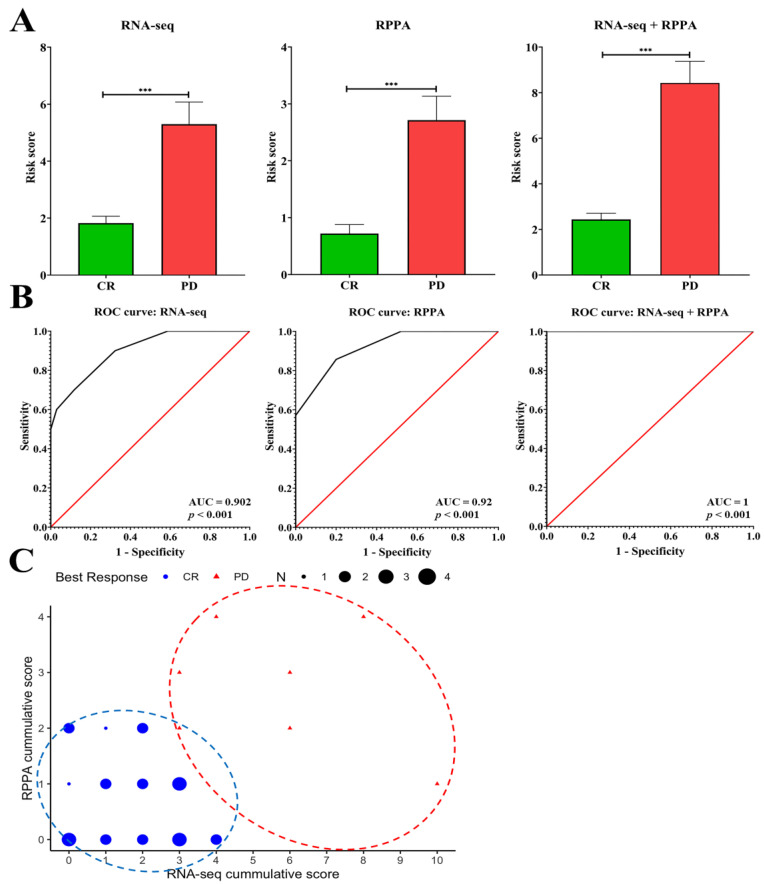
The diagnostic value of the candidate gene set between CR and PD groups. (**A**,**B**) The risk score from RNA-seq, RPPA, and RNA-seq + RPPA analyses showed excellent and outstanding discrimination ability in identifying PD and CR groups. A *p* value less than * *p* < 0.05, ** *p* < 0.01, and *** *p* < 0.001 was considered statistically significant. Red line represents the ROC curve for a line of identity. (**C**) The scatter plot provides a quick view of the co-expression of both RNA-seq and RPPA cumulative risk scores of study cohorts with different clinical outcomes. Blue circles represent the group of CR and red circles represents the group of PD.

We further verified the accuracy of the gene set for distinguishing the PD and CR groups. As mentioned previously, when the values of each target gene reach the cut-off point of high risk, they receive a score of one; otherwise, they receive a score of zero. The sum of the risk scores in the gene set was calculated using the ROC analysis. The results of ROC analysis showed that the gene set of RNA-seq with 10 targets displayed excellent discrimination in identifying PD and CR groups (AUC = 0.902, *p <* 0.001, Figure 3A,B). The gene set of RPPA with five targets also displayed excellent discrimination in identifying PD and CR groups (AUC = 0.92, *p <* 0.001, Figure 3A,B). Moreover, the risk score from the combination of RNA-seq with RPPA with 15 targets obtained outstanding discrimination in identifying PD and CR groups (AUC = 1, *p <* 0.001, Figure 3A,B). The distribution of each subject was further illustrated using a scatter plot with the x-axis of the RNA-seq risk score and the y-axis of the RPPA risk score. The results of the scatter plot showed a clear and separated region between the CR and PD groups (Figure 3C). The above results indicated that the candidate gene set of 15 targets was critical in both SERMs/SERDs and AIs of ET resistance and provided excellent discrimination in identifying PD and CR groups.

### 2.4. The Candidate Gene Set in PFS/OS Outcomes of Treatment Response and Endocrine Therapy Cohort 

**Figure 4:**
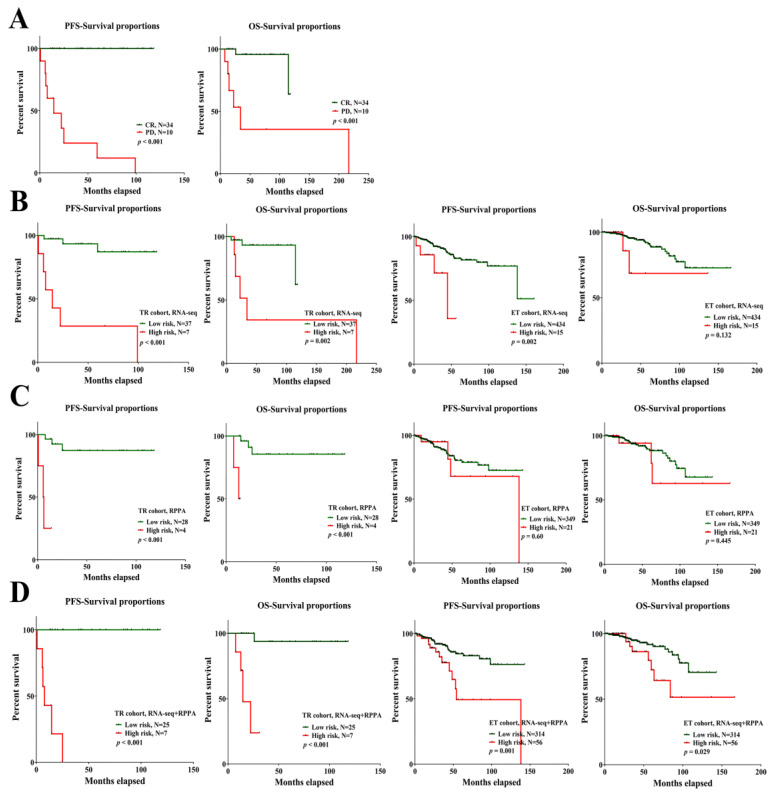
The survival outcomes with cumulative risk score in the treatment response and endocrine therapy cohort. (**A**) The original outcomes of PFS/OS between CR and PD groups. (**B**–**D**) With the mean and median value of the cumulative risk score, the survival outcomes show a significant difference between low- and high-risk groups in the treatment response and endocrine therapy cohort.

The survival outcomes, especially for PFS, were critical clinical points for the evaluation of CR and PD. In the original groups of CR versus PD, the Kaplan–Meier analysis revealed a significant difference in PFS and OS between the CR and PD groups (Figure 4A). To further verify the importance of the candidate gene sets, we evaluated the PFS/OS outcomes using the average and median risk score from Figure 3 (RNA-seq in PD groups: ≥5; RPPA in PD groups: ≥3; RNA-seq + RPPA in PD groups: ≥5) in the treatment response and endocrine therapy cohort. As Figure 4B,C show, based on the cut-off point of the risk score from RNA-seq or RPPA data, the Kaplan–Meier analysis demonstrated a significant difference in PFS and OS between the low-risk and high-risk groups in the treatment response and endocrine therapy cohort (the score from RPPA did not show statistical significance in the endocrine therapy cohort). Moreover, when inputting the risk score from the combination of RNA-seq with RPPA, the Kaplan–Meier analysis also showed a significant difference in PFS and OS between the low-risk and high-risk groups, not only in the treatment response but also in the endocrine therapy cohort (Figure 4D). The above results indicate that the importance of a total of 15 targets was not only observed in the ability to discriminate but also in the outcomes of PFS and OS.

### 2.5. Gene Ontology Analysis (GO) of Candidate Gene Set from PD Groups

**Figure 5:**
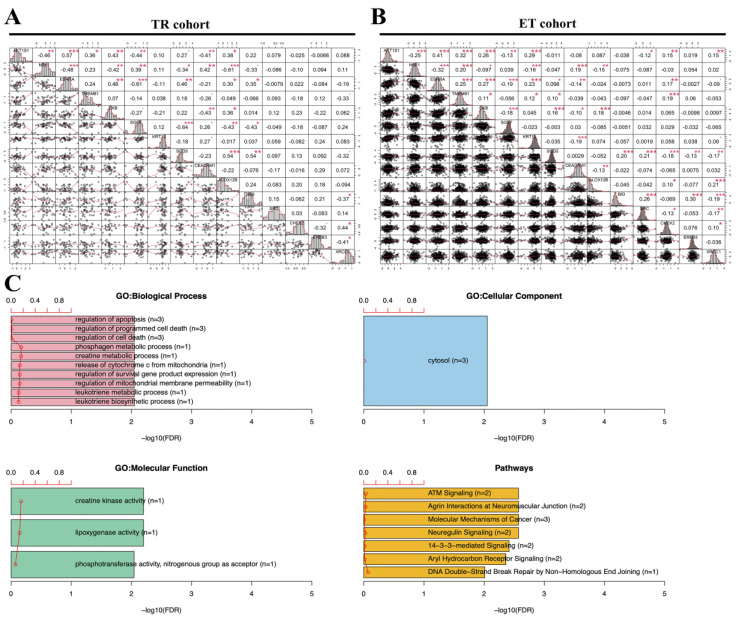
Correlation matrix and GSEA analysis of candidate gene set with 15 targets. (**A**,**B**) Most of the candidate gene sets were significantly correlated with each other in both cohorts. (**C**) The main function of the candidate gene set was associated with cell death, metabolic process, kinase activity, neuregulin, DNA double−strand break repair, and cancer−related signaling pathways. A *p* value less than * *p* < 0.05, ** *p* < 0.01, and *** *p* < 0.001 was considered statistically significant.

The candidate gene set of RNA-seq + RPPA was further analyzed through the correlation matrix and gene set enrichment analysis (GSEA) of gene ontology analysis to determine gene function. From the correlation matrix, we found most of the candidate gene sets were significantly correlated with each other in both cohorts. For example, the high-risk cut-off points for *AKT1S1* were as follows: ≥−0.154, ESRRA: ≥0.281, NSL1: ≤−2.058. The correlation matrix showed that the expression of *AKT1S1* was positively correlated with *ESRRA* (r = 0.57, 0.41) and negatively correlated with *NSL1* (r = −0.46, −0.25) in both cohorts (Figure 5A,B). Thus, the candidate gene sets were not only involved in survival outcomes but were also significantly correlated with each other in both cohorts. Finally, we elucidated the function of candidate gene sets by using the gene ontology analysis, including biological processes, cellular components, molecular functions, and pathways. Regarding biological processes, the candidate gene set was mainly associated with cell death and the metabolic process. Regarding cellular components and molecular functions, the candidate gene set was mainly associated with cytosol and kinase activity. Regarding pathways, the candidate gene set was mainly associated with neuregulin, DNA double-strand break repair, and cancer-related signaling pathways (Figure 5C). The above results provide a potential mechanism and therapeutic strategy for both SERMs/SERDs and AIs of ET resistance in the future. 

## 3. Discussion

In this study, we integrated transcriptomic (RNA-seq) with proteomic (reverse-phase protein array, RPPA) data that appeared in both SERMs/SERDs and AIs from the clinical database of TCGA-BRCA of the GDC data portal to comprehensively analyze the critical targets in endocrine resistance. As previously described, most studies from TCGA mainly focused on the dataset of untreated tumors or datasets without treatment response [18,19,20,21,22]. In our studies, we used two cohorts in which one cohort showed treatment response (treatment response cohort: CR+PD) and the other cohort was without treatment response information but possessed the outcomes of survival (endocrine therapy cohort). In terms of clinical characteristics, the PD groups that were resistant to the first-line ET regimen correlated with a higher proportion of N status and higher stage. Resistance to ET was also correlated with poor PFS and OS. To elucidate the critical targets that appeared in both SERMs/SERDs and AIs from PD groups, a Venn diagram was produced in the region of overlap between SERMs/SERDs and AIs, and among CR, SERMs/SERDs, and AIs.

Regarding the mechanisms of endocrine resistance, loss of ER expression occurs in less than 10% of patients [23,24]. In most cases, endocrine resistance is driven by ligand-independent ER reactivation and is mainly focused on genomic alteration and activation of oncogenic pathways. To date, most studies have concentrated on the mechanisms of genomic alteration. For instance, mutations in ESR1 are observed in approximately 20% of recurrent ER+ breast cancers following long-term treatment with AIs or tamoxifen [9,17]; mutations in oncogenic pathways PI3K (including PIK3CA, PTEN, and AKT1) and MAPK (including NF1, KRAS/NRAS/HRAS, BRAF, and MAP2K1) are also frequently observed and endow endocrine resistance in breast cancer [9,17,25,26]. In addition to genomic alteration, the expression of specific targets is also involved in endocrine resistance; for example, high expression of FGFR1 and c-Myc was associated with tamoxifen resistance [27,28], and overexpression of RAD51 was associated with AI resistance [29]. However, little is known about the specific targets involved in the cross-resistance of ET in breast cancer.

Cross-resistance refers to resistance to several drugs or treatment strategies with a similar mechanism of resistance. In several circumstances, ESR1 mutations confer resistance to AI/SERMs but not to SERD [14], indicating that patients resistant to SERMs with ESR1 mutations may also not benefit from AIs but a clinical benefit from fulvestrant may be yielded [30]. In addition, the loss of NF1 causes resistance to ET in both SERMs and SERDs [25]. To date, few studies have been conducted on the cross-resistance of ET, including SERMs /SERDs and AIs in breast cancer. In this study, we provide the first evidence to elucidate specific targets of transcriptomics and proteomics in the cross-resistance of ET in breast cancer. In the TCGA-BRCA database, the amount of data in RPPA was less than RNA-seq, such that seven targets in the Venn diagram were retained for further study, whereas 1470 mRNA targets were filtered using a series of workflows. Finally, we obtained the optimal gene set of cumulative ROC values for RNA-seq (10 targets) and RPPA (five targets).

From the gene ontology analysis, the candidate gene sets are involved in different categories, including cell death, metabolic process, kinase activity, neuregulin, DNA double-strand break repair, and cancer-related signaling pathways. In these candidate gene sets, CEACAM1 expression is reduced or lost in breast cancer compared to normal tissues and controls the switch of epithelial-to-mesenchymal transition (EMT), which is involved in endocrine resistance [31,32,33,34]. The KRT locus of keratin family, KRT19, is a tumor suppressor gene in breast cancer and regulates drug sensitivity through cancer stem cell reprogramming and NOTCH signaling pathways [35,36,37]. TMEM81 is a transmembrane protein which is involved in the response to fulvestrant treatment [38]. One of the members, TMEM119, has been reported to promote the stemness of breast cancer and is negatively correlated with the survival of patients [39]. ESRRA is overexpressed in a variety of cancers, including breast cancer, and is associated with recurrence, poor prognosis, and tamoxifen/fulvestrant treatment response [40,41,42]. ERBB3 is a typical oncogenic RTK that is upregulated in breast cancer and is directly involved in the development of resistance to both tamoxifen and fulvestrant [43,44,45]. SRC is a non-receptor tyrosine kinase, participating in several oncogenic pathways and promoting tamoxifen resistance in breast cancer [46,47]. AKT1S1 is a substrate of AKT that binds the 14-3-3 protein and is involved in the oncogenic PI3K-Akt pathways, which are critical for endocrine resistance [48,49]. SGEF is a guanine-nucleotide exchange factor which is overexpressed in prostate cancer and its depletion enhanced invadopodia formation in breast cancer [50,51]. SCG5 encodes a neuroendocrine protein and is overexpressed in brain metastatic breast cancer and breast cancer stem cells [52,53]. ALOX12B encodes an enzyme to transfer arachidonic acid to 12R-hydroxyeicosatetraenoic acid. ALOX12B has been reported to promote the carcinogenesis of cervical cancer and is associated with an increased risk of breast cancer [54,55]. CKB is a creatine kinase brain isoform that promotes invasion and metastasis of breast cancer [56]. BID is a pro-apoptotic protein of the Bcl-2 family, participating in drug-induced apoptosis and tamoxifen resistance [57,58]. XRCC1 is a well-known DNA repair gene, and deficiency of XRCC1 promotes an aggressive phenotype of breast cancer [59,60]. NSL1 is a kinetochore-associated protein for cell division, normal development, and accumulation of tumor suppressor gene BRCA1 [61,62]. CHEK2 is also a tumor suppressor gene involved in DNA repair and endocrine resistance [63].

Based on the literature, the function and expression of the candidate gene sets were consistent with our differentiation analysis between CR and PD. For instance, upregulation of ERBB3 in resistance to both tamoxifen and fulvestrant was also consistent with the high expression level in PD groups. Moreover, increased ESRRA expression was associated with both tamoxifen and fulvestrant resistance and was consistent with the high expression level in PD groups. According to the individual optimal cut-off point for RNA-seq and RPPA expression of each target gene, we further showed that scores from the combination of candidate gene sets were higher in PD groups than CR groups, providing excellent discrimination in identifying PD and CR groups. Moreover, the survival outcomes were critical for the therapeutic response in which the PFS/OS was significantly reduced in the endocrine resistance of PD groups. As previously mentioned, most studies from TCGA mainly focused on the dataset without treatment response in which the authors utilize the outcomes of survival to predict ET-resistance-related genes based on the dataset without therapeutic response information [20,21,22]. However, in our studies, we obtained the candidate gene sets from two cohorts with the clinical characteristics of both treatment response and survival outcomes. In our studies, when the average and median scores from PD groups (high risk) were input, we observed a significant difference in PFS/OS not only in the treatment response but also in the endocrine therapy cohort, which indicates that at least 15% of the population may develop endocrine resistance in this cohort. However, the score from RPPA did not reach statistical significance in the endocrine therapy cohort of PFS/OS. The above results may be due to the characteristics of highly heterogeneous factors in endocrine resistance [17]; only five targets in RPPA could not accurately reflect and discriminate the complexity of endocrine resistance, which requires more abundant markers and more functional categories, such as targets in RNA-seq.

The present study still has certain limitations. Due to the limited number of medical records of treatment responses, the number of subjects in the arm of the treatment response cohort was quite small. In the future, we will verify candidate gene sets in a larger number of clinical participants with detailed treatment response records. However, our findings reveal potential targets in the cross-resistance of SERMs/SERDs and AIs based on transcriptomics and proteomics, thereby providing a potential therapeutic approach for endocrine resistance in breast cancer in the future.

## 4. Materials and Methods

### 4.1. Study Population

All data were downloaded from the TCGA-BRCA project in the GDC database using TCGAbiolinks packages. Breast cancer patients who were hormone-positive and had received endocrine therapy (ET) were recruited, and those with incomplete clinical characteristics, RNA sequencing (RNA-seq), and reverse-phase protein array (RPPA) expression profiles were excluded. A total of 44 patients with a record of treatment response (treatment response cohort, TR cohort) and 449 patients (endocrine therapy cohort, ET cohort, without a record of treatment response but with survival outcomes) were analyzed. Both cohorts were used to determine the candidate gene sets and the risk score based on the TR cohort. Baseline clinical characteristics included age at diagnosis, TNM staging, and the pathological stage. The survival endpoints included progression-free survival (PFS) and overall survival (OS).

### 4.2. RNA-Sequencing and Reverse Phase Protein Array (RPPA) Analysis

The RNA-seq expression profile was determined experimentally using the Illumina HiSeq 2000 RNA Sequencing platform at the University of North Carolina TCGA genome characterization center. Differential gene expression (DGE) analysis was conducted to obtain standardized reading count data and to conduct a statistical analysis to identify quantitative changes in gene expression levels based on RNA-seq level 3 data. RNA-seq expression was reported in reads per kilobase million (RPKM), and the current dataset shows the gene-level transcription estimates as log2(x + 1)-transformed RNA-seq by expectation-maximization (RSEM) normalized counts. RPPA is a high-throughput antibody-based technique for protein analysis. RPPA expression was first derived from a single curve using all the samples on a slide with the signal intensity as the response variable and the dilution steps as independent variables. The fitted curve was plotted with the signal intensities on the y-axis and the log2-concentration of proteins on the x-axis for diagnostic purposes. The current dataset abstracted the level 3 RPPA normalized data across all proteins and samples [64]. 

### 4.3. Individual Receiving Operating Characteristics (IROC)

The individual optimal cut-off points for RNA-seq and RPPA expression for each target gene were derived by receiving operating characteristics (ROC) using a treatment response cohort. Each data point was used to dichotomize the study population into high-risk and low-risk groups according to the estimated outcome (initial ET response for TR cohort or survival status for ET cohort). The case numbers of both risk groups and estimated outcome were summarized into four values including true positive (TP), true negative (TN), false negative (FN), and false positive (FP). TP indicates the high-risk subjects with unfavored outcomes (i.e., PD, metastases, or death), TN indicates low-risk subjects with the favored outcome (i.e., CR, disease-free, or survived), FP indicates high-risk subjects with the favored outcome, and FN indicates low-risk subjects with the unfavored outcome. Then, the area under the curve (AUC) of each data point was computed using TP, TN, FP, and FN values based on the area formula as shown in equation [65]. In general, 0.7 ≤ AUC ≤ 0.8 (acceptable discrimination); 0.8 ≤ AUC ≤ 0.9 (excellent discrimination); 0.9 ≤ AUC ≤ 1.0 (outstanding discrimination).

$$\mathrm{AUC}=\frac{\mathrm{TP}}{2\left(\mathrm{TP}+\mathrm{FN}\right)}+\frac{\mathrm{TN}}{2\left(\mathrm{FP}+\mathrm{TN}\right)}$$

The cut-off point that provided the maximized accuracy metric was selected as the optimal cut-off point. An individual AUC (IAUC) of the corresponding optimal cut-off point was computed, and a higher IAUC indicates better predictive ability for the initial ET response. The high-risk characteristic of each target gene was defined by IROC, and an individual risk score of one was scored if the study population obtained high-risk characteristics for the corresponding target genes. In contrast, the individual risk score was zero. 

### 4.4. Cumulative Risk Score and Scatter Plot

Subsequently, the individual risk scores of each target gene were combined to generate a cumulative risk score. The specificity and sensitivity of the risk score from RNA-seq, RPPA, and RNA-seq + RPPA were determined using the ROC curve and AUC value using GraphPad Prism 8 (GraphPad Software, La Jolla, CA, USA). The co-expression of the cumulative risk score based on RNA-seq and RPPA was illustrated using a scatter plot. The x-axis indicates the RNA-seq cumulative risk score, the y-axis indicates the RPPA cumulative risk score, and markers with different shapes and colors indicate different clinical outcomes. Furthermore, the study cohorts were dichotomized into high- and low-risk subgroups based on the mean or median cumulative risk score. Subjects who obtained a cumulative risk score greater than or equal to the mean or median value of PD groups were defined as a high-risk subgroup, and those who obtained a cumulative risk score lower than the mean or median value were defined as low-risk subgroups. The survival difference between the high-risk and low-risk subgroups was estimated to validate the predictive ability of the cumulative risk score for survival outcomes.

### 4.5. Statistical Analysis and Correlation Matrix

Baseline characteristics were summarized as median, range, frequency, and percentage. Differences in baseline characteristics between subgroups were compared using Fisher’s exact and Wilcoxon rank-sum tests. The RNA-seq and RPPA expression of the study cohorts was summarized as median and range, and the difference between groups was estimated using the Wilcoxon rank-sum test. The Venn diagram was conducted based on the criteria of the z-score threshold ± 2.0. The differentiations of the candidate genes in the treatment response cohort were visualized using a box plot. The middle line within the box represents the median and the upper and bottom borders of the box represent the interquartile range. The upper and lower whiskers represent the minimum and maximum values before the fence (Q1/Q3 + 1.5*IQR), and the dots represent the maximum or minimum outliers of the corresponding subgroup. The expression of candidate genes in the treatment response and endocrine therapy cohort was illustrated using a heatmap and annotated with either the treatment response to endocrine therapy or PFS status. The survival rates of the ET response and cumulative risk score subgroups were estimated using the Kaplan–Meier estimator, and the survival difference between subgroups was tested using the log-rank test. Both *p*-values of PFS and OS were estimated using a log-rank test. The distribution and correlation between each target gene were summarized using a correlation matrix, and the correlation between each target gene was tested using the Pearson correlation test. The diagonal shows the histograms for each gene together with the density functions, the lower diagonal shows the scatter plots, and the upper diagonal shows the Pearson correlation coefficients. Moreover, the x-axis indicated the expression of a target from up to down and the y-axis indicated the expression of a target from right to left. All *p*-values were two-tailed, and a *p*-value less than 0.05 was considered statistically significant. All analyses were performed using R 4.0.2 software (R Core Team, 2021) [64] and GraphPad Prism 8 (GraphPad Software, La Jolla, CA, USA).

### 4.6. Gene Set Enrichment Analysis (GSEA)

Gene set enrichment analysis (GSEA) was conducted using the target genes to further explore related gene functions, including biological processes (BP), cellular components (CC), molecular functions (MF), and enrichment pathways. GSEA was performed using the TCGAbiolinks package in R software, and a comprehensive set of efficient and concise annotation tools was derived from the Database for Annotation, Visualization, and Integrated Discovery (DAVID) [66]. The cutoff criterion for GSEA was set at a false discovery rate (FDR) < 0.05 [64].

## 5. Conclusions

In conclusion, we conducted comprehensive transcriptomic (RNA-seq) and proteomic (RPPA) analyses of ET-resistance-related targets from the clinical TCGA-BRCA PanCancer Atlas database. With a series of analyses and selections from both cohorts, we elucidated a candidate gene set of 15 targets that was critical in both SERMs/SERDs and AIs of ET resistance. The candidate gene set provided excellent discrimination in identifying PD and CR groups and was significantly correlated with the survival outcomes of PFS/OS in the treatment response and endocrine therapy cohort. The present study still has certain limitations: (1) some uncertainties and variable factors (different methodologies) exist in the approach with information and computational technology; (2) we lack the time and space characteristics for fully elucidating the interaction and function of targets; (3) we did not take into account the factor of post-translational modification sites (PTMs); (4) there are differences among transcription, translation, and PTM level (not all RNAs are coding for proteins). To overcome the above limitations, we will verify candidate gene sets in a larger number of clinical participants with detailed treatment response records, in vitro cell lines, and in vivo animal models in the future. However, this study provides valuable clinical evidence that candidate gene sets may play a critical role in the diagnosis, mechanism, and therapeutic strategy for both SERMs/SERDs and AIs of ET resistance in the future.

## Figures and Tables

**Table 1:** Baseline distribution of (non-TNBC) study population.

Characteristic	Overall (N = 44)	CR (N = 34)	PD (N = 10)	CR vs. PD, *p*-Value ^a^	PD		CR vs. AI vs. SERM/D, *p*-Value ^b^
					AI (n = 5)	SER (n = 5)	
Age (years)	58 (50, 68)	58 (50, 68)	58 (49, 67)	0.889	61 (45–79)	55 (46–79)	0.917
T stage				0.606			0.444
T1–2	38 (86%)	30 (88%)	8 (80%)		5 (100.0%)	3 (60.0%)	
T3–4	6 (14%)	4 (12%)	2 (20%)		-	2 (40.0%)	
N staging				0.306			1.000
N0	20 (45.5%)	17 (50.0%)	3 (30.0%)		1 (20.0%)	2 (40.0%)	
N1–3	24 (54.5%)	17 (50.0%)	7 (70.0%)		4 (80.0%)	3 (60.0%)	
M stage				0.048			1.000
M0	42 (95%)	34 (100%)	8 (80%)		4 (80.0%)	4 (80.0%)	
M1	2 (4.5%)	0 (0%)	2 (20%)		1 (20.0%)	1 (20.0%)	
Stage				0.043			0.524
Stage I–II	31 (70%)	27 (79%)	4 (40%)		3 (60.0%)	1 (20.0%)	
Stage III–IV	13 (30%)	7 (21%)	6 (60%)		2 (40.0%)	4 (80.0%)	
PFS				<0.001			1.000
Disease-free	35 (80%)	34 (100%)	1 (10%)		1 (20.0%)	-	
Metastases	9 (20%)	0 (0%)	9 (90%)		4 (80.0%)	5 (100.0%)	
OS				<0.001			0.048
Alive	36 (82%)	32 (94%)	4 (40%)		4 (80.0%)	-	
Died	8 (18%)	2 (5.9%)	6 (60%)		1 (20.0%)	5 (100.0%)	
AI	22 (50.0%)	17 (50.0%)	5 (50.0%)	1.000	5 (100%)	-	
SERM	20 (45.5%)	17 (50.0%)	3 (30.0%)	0.306	-	3 (60.0%)	
SERD	2 (4.5%)	0 (0.0%)	2 (20.0%)	0.048	-	2 (40.0%)	

^a^ *p*-value is estimated using the Wilcoxon rank-sum test or Fisher’s exact test. ^b^*p*-value is estimated using the Kruskal–Wallis test or Fisher’s exact test.

**Table 2:** Baseline distribution of treatment response and endocrine therapy cohort in RNA-seq dataset.

Characteristics	Treatment Response Cohort (N = 44)	CR (N = 34)	PD (N = 10)	*p*	Endocrine Therapy Cohort (N = 449)
Age (years)	58 (40–85)	58 (40–85)	58 (45–79)	0.889	61 (49–68)
T staging				0.606	
T1–2	38 (86.4%)	30 (88.2%)	8 (80.0%)		375 (83.5%)
T3–4	6 (13.6%)	4 (11.8%)	2 (20.0%)		74 (16.5%)
N staging				0.306	
N0	20 (45.5%)	17 (50.0%)	3 (30.0%)		214 (47.7%)
N1–3	24 (54.5%)	17 (50.0%)	7 (70.0%)		235 (52.3%)
Stage				0.043	
I–II	31 (70.5%)	27 (79.4%)	4 (40.0%)		333 (74.2%)
III–IV	13 (29.5%)	7 (20.6%)	6 (60.0%)		116 (25.8%)
ET regimen (first-line)					
AI	22 (50.0%)	17 (50.0%)	5 (50.0%)	1.000	235 (52.3%)
SERM	20 (45.5%)	17 (50.0%)	3 (30.0%)	0.306	184 (41.0%)
SERD	2 (4.5%)	0 (0.0%)	2 (20.0%)	0.048	-
Recurrence/Metastases	9 (20.5%)	0 (0.0%)	9 (90.0%)	<0.001	46 (10.2%)
Died	8 (18.2%)	2 (5.9%)	6 (60.0%)	<0.001	29 (6.5%)

*p*-value is estimated using Fisher’s exact test and Wilcoxon rank-sum test.

**Table 3:** Baseline distribution of treatment response and endocrine therapy cohort in RPPA dataset.

Characteristics	Treatment Response Cohort (N = 32)	CR (N = 25)	PD (N = 7)	*p*	Endocrine Therapy Cohort (N = 370)
Age (years)	58 (40–84)	58 (40–84)	55 (45–79)	0.964	61 (49–68)
T staging				0.296	
T1–2	27 (84.4%)	22 (88.0%)	5 (71.4%)		305 (82.4%)
T3–4	5 (15.6%)	3 (12.0%)	2 (28.6%)		65 (17.6%)
N staging				0.678	
N0	17 (53.1%)	14 (56.0%)	3 (42.9%)		167 (45.1%)
N1–3	15 (46.9%)	11 (44.0%)	4 (57.1%)		203 (54.9%)
Stage				0.005	
I–II	24 (75.0%)	22 (88.0%)	2 (28.6%)		267 (72.2%)
III–IV	8 (25.0%)	3 (12.0%)	5 (71.4%)		103 (27.8%)
ET regimen (first- line)					
AI	17 (53.1%)	13 (52.0%)	4 (57.1%)	1.000	189 (51.1%)
SERM	13 (40.6%)	12 (48.0%)	1 (14.3%)	0.195	156 (42.2%)
SERD	2 (6.2%)	0 (0.0%)	2 (28.6%)	0.042	-
Progressed	6 (18.8%)	0 (0.0%)	6 (85.7%)	<0.001	43 (11.6%)
Died	5 (15.6%)	1 (4.0%)	4 (57.1%)	0.004	29 (7.8%)

*p*-value is estimated using Fisher’s exact test and Wilcoxon rank-sum test.

**Table 4:** The results of optimal cut-off point and individual ROC analysis in 29 targets from RNA-seq.

No	Genes	High-Risk	Derivation					Validation			
		Optimal Cut-Off Point	Response	PFS	*p*	OS	*p*	PFS	*p*	OS	*p*
1	SCG5	≥0.555	0.738	0.760	0.005	0.736	0.045	0.502	0.803	0.441	0.062
2	FANK1	≤0.165	0.712	0.690	0.014	0.583	0.511	0.548	0.205	0.592	0.089
3	ALOX12B	≥0.699	0.709	0.721	<0.001	0.604	0.051	0.548	0.120	0.503	0.912
4	CEACAM1	≤−0.862	0.688	0.665	0.017	0.618	0.472	0.572	0.152	0.627	0.053
5	AKT1S1	≥−0.154	0.679	0.700	0.031	0.618	0.282	0.555	0.053	0.485	0.668
6	CDKN1B	≤0.039	0.679	0.622	0.022	0.576	0.039	0.444	0.165	0.431	0.160
7	SDCBP2	≥1.069	0.676	0.805	<0.001	0.771	<0.001	0.500	0.921	0.484	0.467
8	MRPL37	≥0.287	0.668	0.748	0.005	0.639	0.057	0.508	0.628	0.484	0.926
9	ACO2	≥0.264	0.665	0.748	<0.001	0.715	<0.001	0.464	0.586	0.443	0.552
10	PHLDA2	≥1.838	0.662	0.667	<0.001	0.611	<0.001	0.495	0.521	0.495	0.662
11	NSL1	≤−2.058	0.653	0.667	<0.001	0.611	0.002	0.518	0.002	0.512	0.141
12	SLC44A4	≤−0.155	0.653	0.708	<0.001	0.736	<0.001	0.530	0.301	0.533	0.402
13	C9orf68	≤−0.378	0.644	0.665	0.006	0.618	0.036	0.551	0.108	0.603	0.020
14	CALM3	≥1.465	0.644	0.652	<0.001	0.597	0.006	0.512	0.503	0.527	0.164
15	ESRRA	≥0.281	0.644	0.717	0.045	0.771	0.037	0.563	0.035	0.554	0.132
16	NAALADL2	≤−1.431	0.644	0.652	<0.001	0.597	0.004	0.521	0.092	0.529	0.061
17	TMEM81	≥0.744	0.641	0.637	0.039	0.667	0.106	0.559	0.002	0.558	0.006
18	CKB	≥0.464	0.638	0.705	0.027	0.597	0.703	0.575	0.028	0.499	0.887
19	JMJD6	≥−0.082	0.635	0.632	0.109	0.688	0.013	0.438	0.052	0.499	0.758
20	EFNB1	≥0.501	0.632	0.705	0.048	0.750	0.062	0.549	0.126	0.532	0.464
21	CRYBA2	≥−0.086	0.629	0.749	<0.001	0.708	<0.001	0.495	0.975	0.492	0.954
22	HS1BP3	≥2.227	0.626	0.667	<0.001	0.611	0.002	0.495	0.526	0.495	0.646
23	CREG2	≥3.780	0.624	0.667	<0.001	0.611	<0.001	0.494	0.421	0.494	0.573
24	ASPHD1	≥0.850	0.615	0.762	0.001	0.653	0.011	0.523	0.193	0.492	0.681
25	SGEF	≤−3.076	0.615	0.667	<0.001	0.611	<0.001	0.508	0.026	0.496	0.787
26	C12orf35	≤0.378	0.612	0.630	0.071	0.694	0.033	0.561	0.035	0.495	0.543
27	PTPRN	≥1.799	0.603	0.667	<0.001	0.611	<0.001	0.483	0.257	0.483	0.443
28	CNTN5	≥0.945	0.588	0.652	0.013	0.674	0.209	0.534	0.020	0.500	0.793
29	KRT19	≤−2.160	0.559	0.611	<0.001	0.625	<0.001	0.514	0.015	0.509	0.039

*p*-value is estimated using log-rank test.

**Table 5:** The results of optimal cut-off point and individual ROC analysis in seven targets from RPPA.

No	Genes	High-Risk	Derivation					Validation			
		Optimal Cut-off Point	Response	PFS	*p*	OS	*p*	PFS	*p*	OS	*p*
1	BID	≥2.556	0.566	0.667	<0.001	0.581	0.003	0.526	0.170	0.543	0.062
2	CHEK2	≤−0.456	0.646	0.596	0.459	0.652	0.270	0.428	0.067	0.455	0.265
3	ERBB3	≥0.520	0.691	0.718	0.014	0.552	0.323	0.529	0.204	0.557	0.065
4	SERPINE1	≥0.220	0.657	0.641	0.182	0.715	0.076	0.468	0.339	0.437	0.225
5	SRC	≤−0.382	0.571	0.712	0.002	0.644	0.053	0.570	0.010	0.514	0.295
6	STAT5A	≤−0.952	0.611	0.731	0.003	0.544	0.641	0.485	0.387	0.478	0.367
7	XRCC1	≤ −0.627	0.794	0.737	0.008	0.689	0.035	0.491	0.299	0.515	0.771

*p*-value is estimated using log-rank test.

**Table 6:** The RNA-seq expression with reads per kilobase million (RPKM) of candidate genes in the treatment response and endocrine therapy cohort.

Genes	Treatment Response Cohort (N = 44)	CR (N = 34)	PD (N = 10)	*p*	Endocrine Therapy Cohort (N = 449)
AKT1S1	0.08 (−1.44–3.47)	0.00 (−1.44–1.86)	0.23 (−0.15–3.47)	0.090	−0.14 (−2.34–4.51)
NSL1	−0.23 (−2.58–1.99)	−0.07 (−1.61–1.99)	−0.47 (−2.58–1.74)	0.151	0.25 (−3.49–4.00)
ESRRA	0.05 (−1.62–2.48)	0.03 (−1.62–1.70)	0.58 (−1.08–2.48)	0.177	−0.27 (−3.48–2.93)
TMEM81	−0.26 (−1.58–2.52)	−0.37 (−1.58–1.26)	0.32 (−0.98–2.52)	0.186	−0.21 (−3.29–3.43)
CKB	0.21 (−1.72–3.93)	0.15 (−1.72–1.79)	0.57 (−1.61–3.93)	0.196	−0.07 (−2.78–3.55)
SGEF	0.06 (−3.46–1.88)	0.07 (−1.21–1.88)	−0.01 (−3.46–0.71)	0.286	0.28 (−4.19–3.22)
KRT19	0.16 (−4.70–2.40)	0.25 (−1.54–2.40)	0.12 (−4.70−0.86)	0.591	0.19 (−4.65–1.91)
SCG5	0.29 (−2.59–4.03)	0.11 (−2.59–2.19)	0.74 (−0.72–4.03)	0.022	0.00 (−3.81–4.70)
CEACAM1	−0.09 (−2.39–1.53)	0.10 (−1.67–1.53)	−0.58 (−2.39−0.81)	0.075	−0.02 (−3.62–2.17)
ALOX12B	−0.49 (−1.24–3.42)	−0.73 (−1.24–1.13)	0.22 (−1.24–3.42)	0.045	−0.71 (−1.24–5.67)

*p*-value is estimated using Wilcoxon rank-sum test.

**Table 7:** The RPPA expression of candidate genes in the treatment response and endocrine therapy cohort.

Genes	Treatment Response Cohort (N = 32)	CR (N = 25)	PD (N = 7)	*p*	Endocrine Therapy Cohort (N = 370)
BID	0.05 (−0.74, 0.52)	0.04 (−0.72, 0.50)	0.18 (−0.79, 1.59)	0.624	−0.22 (−0.66, 0.39)
SRC	0.13 (−0.29, 0.36)	0.14 (−0.10, 0.35)	−0.20 (−0.43, 0.38)	0.592	−0.10 (−0.46, 0.31)
CHEK2	−0.18 (−0.66, 0.14)	−0.16 (−0.54, 0.13)	−0.46 (−0.99, −0.01)	0.261	−0.12 (−0.51, 0.24)
ERBB3	0.01 (−0.44, 0.56)	−0.06 (−0.41, 0.27)	0.56 (−0.09, 1.11)	0.135	0.01 (−0.40, 0.41)
XRCC1	−0.06 (−0.63, 0.75)	0.24 (−0.48, 0.92)	−0.65 (−2.03, −0.32)	0.018	0.11 (−0.40, 0.68)

*p*-value is estimated using Wilcoxon rank-sum test.

## Data Availability

The data presented in this study are available in this article.

## References

[1] Siegel R.L., Miller K.D., Fuchs H.E., Jemal A. (2022). Cancer statistics, 2022. CA Cancer J. Clin..

[2] Perou C.M., Sorlie T., Eisen M.B., van de Rijn M., Jeffrey S.S., Rees C.A., Pollack J.R., Ross D.T., Johnsen H., Akslen L.A. (2000). Molecular portraits of human breast tumours. Nature.

[3] Pusztai L., Mazouni C., Anderson K., Wu Y., Symmans W.F. (2006). Molecular classification of breast cancer: Limitations and potential. Oncologist.

[4] Hammond M.E., Hayes D.F., Dowsett M., Allred D.C., Hagerty K.L., Badve S., Fitzgibbons P.L., Francis G., Goldstein N.S., Hayes M. (2010). American Society of Clinical Oncology/College of American Pathologists guideline recommendations for immunohistochemical testing of estrogen and progesterone receptors in breast cancer. J. Clin. Oncol..

[5] Nilsson S., Makela S., Treuter E., Tujague M., Thomsen J., Andersson G., Enmark E., Pettersson K., Warner M., Gustafsson J.A. (2001). Mechanisms of estrogen action. Physiol. Rev..

[6] Prall O.W., Rogan E.M., Musgrove E.A., Watts C.K., Sutherland R.L. (1998). c-Myc or cyclin D1 mimics estrogen effects on cyclin E-Cdk2 activation and cell cycle reentry. Mol. Cell. Biol..

[7] Bocchinfuso W.P., Korach K.S. (1997). Mammary gland development and tumorigenesis in estrogen receptor knockout mice. J. Mammary Gland Biol. Neoplasia.

[8] Jordan V.C. (2003). Tamoxifen: A most unlikely pioneering medicine. Nat. Rev. Drug Discov..

[9] Hanker A.B., Sudhan D.R., Arteaga C.L. (2020). Overcoming Endocrine Resistance in Breast Cancer. Cancer Cell.

[10] Cuzick J., Sestak I., Baum M., Buzdar A., Howell A., Dowsett M., Forbes J.F., the ATAC/LATTE investigators (2010). Effect of anastrozole and tamoxifen as adjuvant treatment for early-stage breast cancer: 10-year analysis of the ATAC trial. Lancet Oncol..

[11] Men X., Ma J., Wu T., Pu J., Wen S., Shen J., Wang X., Wang Y., Chen C., Dai P. (2018). Transcriptome profiling identified differentially expressed genes and pathways associated with tamoxifen resistance in human breast cancer. Oncotarget.

[12] Haque M.M., Desai K.V. (2019). Pathways to Endocrine Therapy Resistance in Breast Cancer. Front. Endocrinol..

[13] Pan H., Gray R., Braybrooke J., Davies C., Taylor C., McGale P., Peto R., Pritchard K.I., Bergh J., Dowsett M. (2017). 20-Year Risks of Breast-Cancer Recurrence after Stopping Endocrine Therapy at 5 Years. N. Engl. J. Med..

[14] Toy W., Shen Y., Won H., Green B., Sakr R.A., Will M., Li Z., Gala K., Fanning S., King T.A. (2013). ESR1 ligand-binding domain mutations in hormone-resistant breast cancer. Nat. Genet..

[15] Nayar U., Cohen O., Kapstad C., Cuoco M.S., Waks A.G., Wander S.A., Painter C., Freeman S., Persky N.S., Marini L. (2019). Acquired HER2 mutations in ER(+) metastatic breast cancer confer resistance to estrogen receptor-directed therapies. Nat. Genet..

[16] Miller T.W., Balko J.M., Fox E.M., Ghazoui Z., Dunbier A., Anderson H., Dowsett M., Jiang A., Smith R.A., Maira S.M. (2011). ERalpha-dependent E2F transcription can mediate resistance to estrogen deprivation in human breast cancer. Cancer Discov..

[17] Razavi P., Chang M.T., Xu G., Bandlamudi C., Ross D.S., Vasan N., Cai Y., Bielski C.M., Donoghue M.T.A., Jonsson P. (2018). The Genomic Landscape of Endocrine-Resistant Advanced Breast Cancers. Cancer Cell..

[18] Ciriello G., Gatza M.L., Beck A.H., Wilkerson M.D., Rhie S.K., Pastore A., Zhang H., McLellan M., Yau C., Kandoth C. (2015). Comprehensive Molecular Portraits of Invasive Lobular Breast Cancer. Cell.

[19] Nik-Zainal S., Davies H., Staaf J., Ramakrishna M., Glodzik D., Zou X., Martincorena I., Alexandrov L.B., Martin S., Wedge D.C. (2016). Landscape of somatic mutations in 560 breast cancer whole-genome sequences. Nature.

[20] Zhang F., Cui Y. (2019). Dysregulation of DNA methylation patterns may identify patients with breast cancer resistant to endocrine therapy: A predictive classifier based on differentially methylated regions. Oncol. Lett..

[21] Soleimani Dodaran M., Borgoni S., Sofyali E., Verschure P.J., Wiemann S., Moerland P.D., van Kampen A.H.C. (2020). Candidate methylation sites associated with endocrine therapy resistance in ER+/HER2- breast cancer. BMC Cancer.

[22] Shuai C., Yuan F., Liu Y., Wang C., Wang J., He H. (2021). Estrogen receptor-positive breast cancer survival prediction and analysis of resistance-related genes introduction. PeerJ.

[23] Ellis M.J., Tao Y., Luo J., A’Hern R., Evans D.B., Bhatnagar A.S., Chaudri Ross H.A., von Kameke A., Miller W.R., Smith I. (2008). Outcome prediction for estrogen receptor-positive breast cancer based on postneoadjuvant endocrine therapy tumor characteristics. J. Natl. Cancer Inst..

[24] Shiino S., Kinoshita T., Yoshida M., Jimbo K., Asaga S., Takayama S., Tsuda H. (2016). Prognostic Impact of Discordance in Hormone Receptor Status between Primary and Recurrent Sites in Patients with Recurrent Breast Cancer. Clin. Breast Cancer.

[25] Pearson A., Proszek P., Pascual J., Fribbens C., Shamsher M.K., Kingston B., O’Leary B., Herrera-Abreu M.T., Cutts R.J., Garcia-Murillas I. (2020). Inactivating NF1 Mutations Are Enriched in Advanced Breast Cancer and Contribute to Endocrine Therapy Resistance. Clin. Cancer Res..

[26] Chien T.J. (2021). A review of the endocrine resistance in hormone-positive breast cancer. Am. J. Cancer Res..

[27] Lv Q., Guan S., Zhu M., Huang H., Wu J., Dai X. (2021). FGFR1 Is Associated with Tamoxifen Resistance and Poor Prognosis of ER-Positive Breast Cancers by Suppressing ER Protein Expression. Technol. Cancer Res. Treat..

[28] Chen R., Guo S., Yang C., Sun L., Zong B., Li K., Liu L., Tu G., Liu M., Liu S. (2020). Although cMYC contributes to tamoxifen resistance, it improves cisplatin sensitivity in ERpositive breast cancer. Int. J. Oncol..

[29] Jia Y., Song Y., Dong G., Hao C., Zhao W., Li S., Tong Z. (2019). Aberrant Regulation of RAD51 Promotes Resistance of Neoadjuvant Endocrine Therapy in ER-positive Breast Cancer. Sci. Rep..

[30] Perey L., Paridaens R., Hawle H., Zaman K., Nole F., Wildiers H., Fiche M., Dietrich D., Clement P., Koberle D. (2007). Clinical benefit of fulvestrant in postmenopausal women with advanced breast cancer and primary or acquired resistance to aromatase inhibitors: Final results of phase II Swiss Group for Clinical Cancer Research Trial (SAKK 21/00). Ann. Oncol..

[31] Yang C., He P., Liu Y., He Y., Yang C., Du Y., Zhou M., Wang W., Zhang G., Wu M. (2015). Down-regulation of CEACAM1 in breast cancer. Acta Biochim. Biophys. Sin..

[32] Wegwitz F., Lenfert E., Gerstel D., von Ehrenstein L., Einhoff J., Schmidt G., Logsdon M., Brandner J., Tiegs G., Beauchemin N. (2016). CEACAM1 controls the EMT switch in murine mammary carcinoma in vitro and in vivo. Oncotarget.

[33] Gooding A.J., Schiemann W.P. (2020). Epithelial-Mesenchymal Transition Programs and Cancer Stem Cell Phenotypes: Mediators of Breast Cancer Therapy Resistance. Mol. Cancer Res..

[34] Vesuna F., Lisok A., Kimble B., Domek J., Kato Y., van der Groep P., Artemov D., Kowalski J., Carraway H., van Diest P. (2012). Twist contributes to hormone resistance in breast cancer by downregulating estrogen receptor-alpha. Oncogene.

[35] Saha S.K., Kim K., Yang G.M., Choi H.Y., Cho S.G. (2018). Cytokeratin 19 (KRT19) has a Role in the Reprogramming of Cancer Stem Cell-Like Cells to Less Aggressive and More Drug-Sensitive Cells. Int. J. Mol. Sci..

[36] Saha S.K., Choi H.Y., Kim B.W., Dayem A.A., Yang G.M., Kim K.S., Yin Y.F., Cho S.G. (2017). KRT19 directly interacts with beta-catenin/RAC1 complex to regulate NUMB-dependent NOTCH signaling pathway and breast cancer properties. Oncogene.

[37] Ju J.H., Yang W., Lee K.M., Oh S., Nam K., Shim S., Shin S.Y., Gye M.C., Chu I.S., Shin I. (2013). Regulation of cell proliferation and migration by keratin19-induced nuclear import of early growth response-1 in breast cancer cells. Clin. Cancer Res..

[38] Jeselsohn R., Barry W.T., Migliaccio I., Biagioni C., Zhao J., De Tribolet-Hardy J., Guarducci C., Bonechi M., Laing N., Winer E.P. (2016). TransCONFIRM: Identification of a Genetic Signature of Response to Fulvestrant in Advanced Hormone Receptor-Positive Breast Cancer. Clin. Cancer Res..

[39] Yang B., Wang F., Zheng G. (2021). Transmembrane protein TMEM119 facilitates the stemness of breast cancer cells by activating Wnt/beta-catenin pathway. Bioengineered.

[40] Suzuki T., Miki Y., Moriya T., Shimada N., Ishida T., Hirakawa H., Ohuchi N., Sasano H. (2004). Estrogen-related receptor alpha in human breast carcinoma as a potent prognostic factor. Cancer Res..

[41] Manna S., Bostner J., Sun Y., Miller L.D., Alayev A., Schwartz N.S., Lager E., Fornander T., Nordenskjold B., Yu J.J. (2016). ERRalpha Is a Marker of Tamoxifen Response and Survival in Triple-Negative Breast Cancer. Clin. Cancer Res..

[42] Thewes V., Simon R., Schroeter P., Schlotter M., Anzeneder T., Buttner R., Benes V., Sauter G., Burwinkel B., Nicholson R.I. (2015). Reprogramming of the ERRalpha and ERalpha target gene landscape triggers tamoxifen resistance in breast cancer. Cancer Res..

[43] Ma J., Lyu H., Huang J., Liu B. (2014). Targeting of erbB3 receptor to overcome resistance in cancer treatment. Mol. Cancer.

[44] Liu B., Ordonez-Ercan D., Fan Z., Edgerton S.M., Yang X., Thor A.D. (2007). Downregulation of erbB3 abrogates erbB2-mediated tamoxifen resistance in breast cancer cells. Int. J. Cancer.

[45] Hutcheson I.R., Goddard L., Barrow D., McClelland R.A., Francies H.E., Knowlden J.M., Nicholson R.I., Gee J.M. (2011). Fulvestrant-induced expression of ErbB3 and ErbB4 receptors sensitizes oestrogen receptor-positive breast cancer cells to heregulin beta1. Breast Cancer Res..

[46] Larsen S.L., Laenkholm A.V., Duun-Henriksen A.K., Bak M., Lykkesfeldt A.E., Kirkegaard T. (2015). SRC drives growth of antiestrogen resistant breast cancer cell lines and is a marker for reduced benefit of tamoxifen treatment. PLoS ONE.

[47] Zhou J., Xu M., Le K., Ming J., Guo H., Ruan S., Huang T. (2020). SRC Promotes Tamoxifen Resistance in Breast Cancer via Up-Regulating SIRT1. Onco Targets Ther..

[48] Huang B., Porter G. (2005). Expression of proline-rich Akt-substrate PRAS40 in cell survival pathway and carcinogenesis. Acta Pharmacol. Sin..

[49] Bostner J., Alayev A., Berman A.Y., Fornander T., Nordenskjold B., Holz M.K., Stal O. (2018). Raptor localization predicts prognosis and tamoxifen response in estrogen receptor-positive breast cancer. Breast Cancer Res. Treat..

[50] Wang H., Wu R., Yu L., Wu F., Li S., Zhao Y., Li H., Luo G., Wang J., Zhou J. (2012). SGEF is overexpressed in prostate cancer and contributes to prostate cancer progression. Oncol. Rep..

[51] Goicoechea S.M., Zinn A., Awadia S.S., Snyder K., Garcia-Mata R. (2017). A RhoG-mediated signaling pathway that modulates invadopodia dynamics in breast cancer cells. J. Cell Sci..

[52] Mamoor S. (2021). SCG5 Is a Differentially Expressed Gene in Human Metastatic Breast Cancer, in the Brain and in the Lymph Nodes.

[53] Colacino J.A., Azizi E., Brooks M.D., Harouaka R., Fouladdel S., McDermott S.P., Lee M., Hill D., Madden J., Boerner J. (2018). Heterogeneity of Human Breast Stem and Progenitor Cells as Revealed by Transcriptional Profiling. Stem Cell Rep..

[54] Jiang T., Zhou B., Li Y.M., Yang Q.Y., Tu K.J., Li L.Y. (2020). ALOX12B promotes carcinogenesis in cervical cancer by regulating the PI3K/ERK1 signaling pathway. Oncol. Lett..

[55] Lee J.Y., Park A.K., Lee K.M., Park S.K., Han S., Han W., Noh D.Y., Yoo K.Y., Kim H., Chanock S.J. (2009). Candidate gene approach evaluates association between innate immunity genes and breast cancer risk in Korean women. Carcinogenesis.

[56] Krutilina R.I., Playa H., Brooks D.L., Schwab L.P., Parke D.N., Oluwalana D., Layman D.R., Fan M., Johnson D.L., Yue J. (2021). HIF-Dependent CKB Expression Promotes Breast Cancer Metastasis, Whereas Cyclocreatine Therapy Impairs Cellular Invasion and Improves Chemotherapy Efficacy. Cancers.

[57] Meresman G.F., Bilotas M., Abello V., Buquet R., Tesone M., Sueldo C. (2005). Effects of aromatase inhibitors on proliferation and apoptosis in eutopic endometrial cell cultures from patients with endometriosis. Fertil. Steril..

[58] Yin H., Zhu Q., Liu M., Tu G., Li Q., Yuan J., Wen S., Yang G. (2017). GPER promotes tamoxifen-resistance in ER+ breast cancer cells by reduced Bim proteins through MAPK/Erk-TRIM2 signaling axis. Int. J. Oncol..

[59] Sultana R., Abdel-Fatah T., Abbotts R., Hawkes C., Albarakati N., Seedhouse C., Ball G., Chan S., Rakha E.A., Ellis I.O. (2013). Targeting XRCC1 deficiency in breast cancer for personalized therapy. Cancer Res..

[60] Abdel-Fatah T.M., Perry C., Arora A., Thompson N., Doherty R., Moseley P.M., Green A.R., Chan S.Y., Ellis I.O., Madhusudan S. (2014). Is there a role for base excision repair in estrogen/estrogen receptor-driven breast cancers?. Antioxid. Redox Signal..

[61] Sheikh B.N., Guhathakurta S., Akhtar A. (2019). The non-specific lethal (NSL) complex at the crossroads of transcriptional control and cellular homeostasis. EMBO Rep..

[62] Altiok S., Batt D., Altiok N., Papautsky A., Downward J., Roberts T.M., Avraham H. (1999). Heregulin induces phosphorylation of BRCA1 through phosphatidylinositol 3-Kinase/AKT in breast cancer cells. J. Biol. Chem..

[63] Haricharan S., Punturi N., Singh P., Holloway K.R., Anurag M., Schmelz J., Schmidt C., Lei J.T., Suman V., Hunt K. (2017). Loss of MutL Disrupts CHK2-Dependent Cell-Cycle Control through CDK4/6 to Promote Intrinsic Endocrine Therapy Resistance in Primary Breast Cancer. Cancer Discov..

[64] Kan J.Y., Moi S.H., Hung W.C., Hou M.F., Chen F.M., Shih S.L., Shiau J.P., Li C.L., Chiang C.P. (2020). Comprehensive Transcriptomic Analysis Identifies ST8SIA1 as a Survival-Related Sialyltransferase Gene in Breast Cancer. Genes.

[65] Pontius R.G., Parmentier B. (2014). Recommendations for using the relative operating characteristic (ROC). Landsc. Ecol..

[66] Huang D.W., Sherman B.T., Lempicki R.A. (2009). Systematic and integrative analysis of large gene lists using DAVID bioinformatics resources. Nat. Protoc..

